# Construction of a Molecular Dynamics Model of N-A-S-H Geopolymer Based on XRD Analysis

**DOI:** 10.3390/ma17246103

**Published:** 2024-12-13

**Authors:** Qing Wang, Hewei Li, Zhaoyang Ding, Rui Shan, Mingyu Zhao

**Affiliations:** 1 College of Materials Science and Engineering, Shenyang Jianzhu University, Shenyang 110168, Chinalehe0739@stu.sjzu.edu.cn (H.L.); zmyzhaomingyu@sjzu.edu.cn (M.Z.); 2 National Engineering Research Center of Building Technology, Beijing 101100, China

**Keywords:** geopolymer, N-A-S-H, XRD, molecular dynamics, simulation

## Abstract

A geopolymer is a low-carbon cementitious material, and its condensation process is akin to the formation of inorganic polymers. The crystal phase of synthesized geopolymers was identified using XRD; the scattering peaks of amorphous phases were analyzed, and the zeolite minerals akin to different n(Si)/n(Al) geopolymers were determined. Based on this, a model structure of N-A-S-H geopolymers was established. The molecular dynamics structure of the model was simulated, and the density, energy, and bulk modulus of the model were calculated using three different force fields. According to the calculation results, the most suitable force field for N-A-S-H calculation is COMPASS III. In this study, all calculations were performed using MaterialsStudio 7.0. The research process introduces a new modeling method for geopolymers, similar to building C-S-H based on Tobermorite, which aids in advancing the molecular dynamics simulation of geopolymers.

## 1. Introduction

It has been more than 40 years since the development of geopolymers [1]. In 1978, French scientist J. Davidovits [2] first published an invention patent under the name ‘geopolymer’ in his research on Ancient Roman architecture. In 1982, the world’s first geopolymer binder MK-750 was invented—an inorganic polymer formed by calcining kaolin at 750 °C and then activating it with potassium hydroxide or sodium hydroxide. Nowadays, a geopolymer is defined as a kind of cementitious material formed by the action of an alkaline solution using natural minerals with hydraulic characteristics or low-calcium solid waste as raw materials (such as fly ash [3,4], red mud [5], metakaolin [6,7], nanometer silica [8], etc.) [9]. Compared with traditional cement, the production process of geopolymers recycles a large amount of industrial waste, meeting the requirements of the current environmental protection era [10], and has the advantages of being green and low-carbon [11]. However, due to the wide sources of geopolymer precursors, diverse activation methods, and complex hardening processes, the properties of geopolymers are unstable and their type discreteness is stronger than that of cement. Undoubtedly, the research on geopolymers is full of opportunities and challenges [12].

Different from the hydration reaction of cement by a clear material mineral to form hydrated calcium silicate gel (CaO-SiO_2_-H_2_O, referred to as C-S-H), the condensation process of cementitious geopolymer material is similar to the condensation method of polymer [13]. Davidovits proposed that the basic structural monomers of geopolymers are single silicon–aluminum monomer PS (-Si-O-Al-O-), double silicon–aluminum monomer PSS (-Si-O-Al-O-Si-O-), and three silicon–aluminum monomer PSDS (-Si-O-Al-O-Si-O-Si-O-Si-O-) [14]. The silicon–aluminum oxide generates unstable PS, PSS, and PSDS under the influence of alkaline solution and forms a three-dimensional network structure by bridging oxygen condensation to form a three-dimensional aluminosilicate mineral polymer containing a variety of amorphous to semi-crystalline phases [15]. For the condensation process, the theory of covalent bonding is generally recognized by scholars [16]. The theory is based on alkali-activated metakaolin as an example [17]. Under the action of alkali activation, the silicon–oxygen in the metakaolin is dissociated to form a variety of orthosilicate aluminate monomers (PS) [18], which are dehydrated and condensed into a hydroxy sodalite network structure in an alkaline environment [19]. In recent years, the condensation theory of geopolymers has been deeply studied. It is generally believed that the alkali-activated process of aluminosilicate minerals is divided into three stages: the dissolution stage, which makes the aluminosilicate oxide obtain chemical activity; then the stage of forming amorphous hydration products, in which the dissolved Al^3+^ and Si^4+^ react with the positive ions in the activator to form a hydration product with a three-dimensional amorphous structure [M_x_(AlO_2_)_y_(SiO_2_)_x_·nNaOH·mH_2_O]. In the hardening stage of dehydration condensation, dehydration condensation occurs between the formed aluminum oxide monomer and the silicon oxide monomer, and finally polymerizes into a hydrated sodium aluminosilicate gel (Na_2_O-Al_2_O_3_-SiO_2_-H_2_O, referred to as N-A-S-H) [20]. Since the main structure of N-A-S-H is an amorphous gel, it is difficult to quantitatively characterize the microstructure of the gel by conventional methods and systematically study it [21].

Molecular Dynamics (MD) is an approach in computational materials science. The origins of MD can be traced back to 1956 when Alder and Wainwright introduced a computer simulation method for studying the dynamics of hard sphere assembly using macroscopic models to mimic microscopic phenomena [22]. Over the past 60 years, MD has found extensive applications in materials science, biology, and chemistry. Particularly in the last decade, MD has evolved into a powerful analytical tool for constructing cementitious materials [23]. The cornerstone of MD research lies in the development of molecular models. Guan et al. [24] utilized seven types of silicon–aluminum chains with varying silicon–aluminum ratios and configurations as structural units. Monte Carlo treatment was applied to each structural unit, and Na was inserted in equal numbers to Al. An MD simulation of melting–quenching was conducted to establish the molecular model of N-A-S-H gel, successfully achieving dehydration condensation between monomers. Sadat et al. [25] replaced Si atoms randomly with Al atoms in a pure four-coordinated Si environment and placed Na atoms around the Al atoms. The model was quenched and heated to 2500K for a duration before rapidly cooling to 300K to obtain the sodium aluminosilicate glass model. Gideon et al. [26] segmented sodium aluminosilicate into upper and lower layers, introduced water molecules between the layers, and used MD to simulate N-A-S-H. Lolli et al. [27] employed MD to construct N-A-S-H based on the sodalite cage, creating defects by deleting atoms, substituting Si with Al, and adding corresponding Na and H_2_O. The model’s accuracy can be validated by comparing the simulation model information with real samples prepared in the laboratory. Ideal verification data, such as PDF [28], XRD [29], and Young’s modulus [30], are recorded for this purpose.

This paper introduces a modeling method for geopolymers. Rietveld refinement analysis was performed on geopolymers with different silicon–aluminum ratios (n(Si)/n(Al)). The crystal diffraction peaks were removed to obtain the scattering peaks of the amorphous phase. Based on the fitting results of the scattering peaks of the amorphous phase, geopolymer models with different silicon–aluminum ratios were constructed. Subsequently, MaterialsStudio 7.0 was used to perform dynamic relaxation under three force fields to calculate the density, energy, and elastic modulus of the model. The molecular dynamics force field suitable for the geopolymer model was determined.

## 2. Materials and Experimental Methods

### 2.1. Raw Materials

To mitigate the interference of various metal cations from industrial waste on the test results, this paper selected metakaolin and fly ash with high reactivity for the preparation of geopolymers. In this study, the raw materials were calcined at 750 °C for metakaolin (MK, Shijiazhuang City, China) as the matrix material, and the n(Si)/n(Al) ratio was adjusted using 98% active nanometer silica (NS, Zhengzhou City, China). The chemical properties of MK and NS were analyzed by X-ray fluorescence spectrometer (S2 RANGER), Bruker AXS Company, Karlsruhe, Germany (Table 1). Analytically pure sodium hydroxide was dissolved in a 10 M concentration aqueous solution as an alkali activator for the activation and preparation of geopolymers. The composition ratio of each component precursor material is shown in Table 2, where n(Si)/n(Al) is determined by Equation (1), where i represents metakaolin and nanometer silica.

(1) $$n(\mathrm{Si})/n(\mathrm{Al})=\frac{\sum m_{i}\times \omega_{(i,{\mathrm{SiO}}_{2})}/60.084}{2\times \sum m_{i}\times \omega_{(i,{\mathrm{Al}}_{2}O_{3})}/101.96}$$

### 2.2. X-Ray Data Acquisition and Analysis

The samples were placed in an oven at 80 °C for drying until a constant weight was achieved. At this temperature, the adsorbed water in the geopolymer can be effectively evaporated without significantly affecting the chemically bound water. After being fully ground with an agate mortar, the samples were manually sieved with a 75 μm sieve, and the mixed samples were filled into the quartz sample tank using the back pressure method. To prevent moisture and CO_2_ in the air from affecting the results, it was required that the powder be exposed to the air for no more than 15 min before analysis. The QXRD analysis was conducted using a XRD-700 X-ray diffractometer, Shimadzu, Kyoto, Japan. The equipment utilized CuKα radiation, with an operating voltage of 40 kV and a tube current of 30 mA. The analysis employed a step scanning method with a step size of 0.02°, a counting time of 2 s for each step, and a diffraction angle range of 10° to 50°.

**Figure 1:**
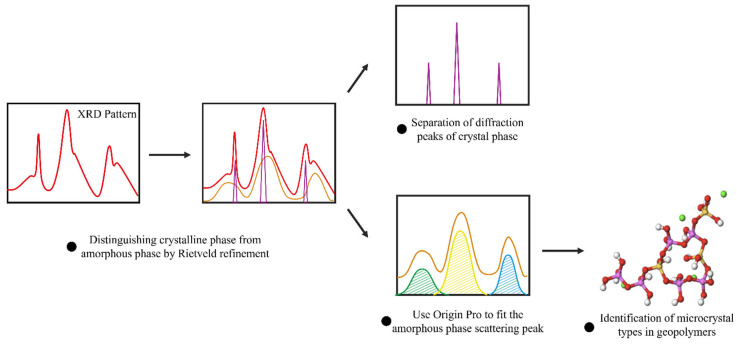
Identification results of amorphous phase scattering peaks.

The method of crystal phase determination through refinement is illustrated in Figure 1. Highscore 5.2 was utilized to identify the crystal phase composition, while GSAS-II [31] software was employed for Rietveld refinement of the XRD pattern. To ensure the accuracy of the refinement, the fitting value (Rwp) needed to be reduced to approximately 10% for it to be deemed effective. If the Rwp cannot be decreased after several cycles of operation, the crystal phase is re-refined. Once the complete crystal phase information is obtained, it is isolated, and only the background part of the refinement fitting is retained. This section consists of window scattering and scattering peaks of amorphous materials. The window scattering interference is eliminated by leveling the baseline to isolate the scattering peaks of amorphous components. Peak fitting was conducted using Origin Pro, 9.0. The diffraction peak positions from standard card data of natural minerals were used as the peak center sites. The Voigt peak function was applied for fitting, and the result with the highest fitting value was chosen as the final outcome.

### 2.3. Simulation Model and Methodology

The Forcite module of MaterialsStudio 7.0 was used to optimize the energy minimization structure of the model. Universal, COMPASS III, and ClayFF force fields were selected. Quality was always set to Fine, Algorithm was set to Smart, and Electrostatic and van der Waals were set to Atom-based. Then, the Anneal calculation of the canonical ensemble (NVT) of the box was carried out. The initial temperature was 300 K, which was then raised to 500 K, and the heating cycle was repeated five times, each time running for 100 ps. After the operation, the adsorption module was used to adsorb molecular water.

## 3. Results and Discussion

### 3.1. Approximate Structure of N-A-S-H Geopolymers

**Figure 2:**
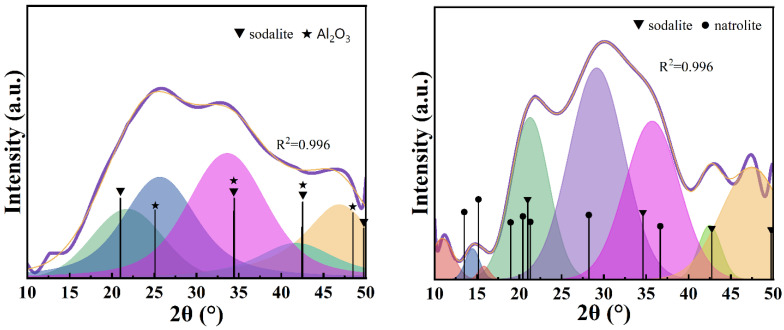
Optimal results of peak fitting of GEO-1 (**left**) and GEO-2 (**right**) amorphous phases.

After fitting the scattering peaks of the amorphous phase of GEO-1 (Figure 2, left), it was found that the amorphous phase of GEO-1 had the best matching effect with the characteristic peaks of sodalite and corundum, and the fitting value reached 0.996. The hydrated sodalite is expressed as Na_6_(AlSiO_4_)_6_(H_2_O)_8_, which belongs to zeolite minerals and is called the ‘hydrosodalite’ group of minerals (the commonly reported hydrosodalite has basic sodalite and hydroxysodalite, and the chemical structures of the two substances are slightly different). The amorphous gel composition can be written as Na_6_[(Si-O_2_)_1_-Al-O_2_]_6_·8H_2_O, satisfying Davidovits’ opinion [2] that the general molecular formula of the N-A-S-H structure is Na_n_[(Si-O_2_)_Z_-Al-O_2_]_n_·wH_2_O. Al_2_O_3_ is composed of aluminum components that fail to enter the silicon–aluminum network structure. The amorphous phase of GEO-1 is composed of sodalite and Al_2_O_3_, so the actual n(Si)/n(Al) should be less than 1. After fitting the scattering peaks of the amorphous phase of GEO-2 (Figure 2, right), it was found that the amorphous phase of GEO-2 had the best matching effect with the characteristic peaks of sodalite and natrolite, and the fitting value reached 0.996. The chemical expression of natrolite is Na_2_Al_2_Si_3_O_10_(H_2_O)_2_, and its amorphous gel composition can be written as Na_2_[(Si-O_2_)_1.5_-Al-O_2_]_2_·2H_2_O, which satisfies the general molecular formula of the N-A-S-H structure proposed by Davidovits [2]. The amorphous phase of GEO-2 is composed of sodalite and natrolite, so the actual n(Si)/n(Al) should be between 1 and 1.5. The n(Si)/n(Al) of amorphous GEO-1 and GEO-2 phases are smaller than that of the powder material, which is related to the fact that metakaolin contains some insoluble quartz.

**Figure 3:**
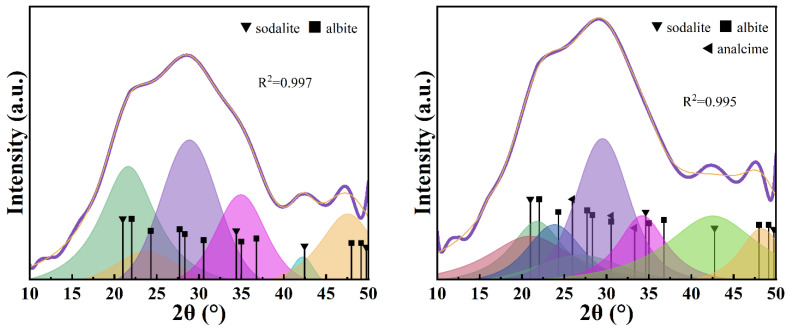
Optimal results of peak fitting of GEO-3 (**left**) and GEO-4 (**right**) amorphous phases.

After fitting the scattering peaks of the amorphous phase of GEO-3 (Figure 3, left), it was found that the amorphous phase of GEO-3 had the best matching effect with the characteristic peaks of sodalite and albite, and the fitting value reached 0.997. The chemical formula of albite is Na(AlSi_3_O_8_), and its amorphous hydrogel composition can be written as Na_2_[(Si-O_2_)_3_-Al-O_2_]_2_·wH_2_O, which satisfies the general molecular formula of the N-A-S-H structure. The amorphous phase of GEO-3 is composed of sodalite and albite, so the actual n(Si)/n(Al) ratio should be between 1 and 3. After fitting the scattering peaks of the amorphous phase of GEO-4 (Figure 3, right), it was found that the amorphous phase of GEO-4 had the best matching effect with the characteristic peaks of sodalite, analcime, and albite, and the fitting value reached 0.995. The chemical formula of analcime is NaAlSi_2_O_6_·H_2_O, and its amorphous gel composition can be written as Na_1_[(Si-O_2_)_2_-Al-O_2_]_1_·1H_2_O, which satisfies the general molecular formula of the N-A-S-H structure. The amorphous phase of GEO-4 is composed of sodalite, analcime, and albite, so the actual n(Si)/n(Al) ratio should be between 1 and 3.

In summary, the basic structural units of geopolymers are close to various types of aluminosilicate six-membered ring zeolites, and often form products similar to sodalite. Similarly, scholars often use sodium hydroxide to treat silicon–aluminum powders to obtain the above-mentioned zeolites. Although synthetic zeolites require a higher reaction temperature, the reaction mechanisms in these two cases are similar. Jae et al. [32] obtained hydroxysodalite by NaOH-activated fly ash at 90 °C and atmospheric pressure, and considered that hydroxysodalite is the best candidate to represent the intermediate structure of the synthetic zeolite process. The role of hydroxysodalite in geopolymer research is similar to the role of 14 Å Tobermorite and Perlite in C-S-H [33]. The zeolite phases detected in this paper are close to the arrangement of sodalite, all of which are hexagonal silicon aluminum ring structure of ABC-6 family, which is in line with the formation mechanism of geopolymer. The difference is that the size of n(Si)/n(Al) is different. Therefore, in order to realize the construction of a geopolymer model, the crystal model of the above minerals is selected as the basis, and the N-A-S-H model is built by referring to the method of building a C-S-H model based on 14 Å Tobermorite and Perlite. In addition, nepheline is often reported in geopolymers with n(Si)/n(Al) = 1 [34], which is also built in this paper.

### 3.2. Modeling of the N-A-S-H Geopolymer Model

It can be seen from the above that the N-A-S-H structure of the geopolymer is close to that of the ABC-6 family zeolite, and different products are formed due to the difference in n(Si)/n(Al). The N-A-S-H model of different n(Si)/n(Al) was constructed based on a variety of ABC-6 family zeolites by molecular dynamics simulation. The specific steps of using MaterialsStudio 7.0 to build the N-A-S-H model based on ABC-6 family zeolite are as follows:

(1) In the American Mineralogist Crystal Structure Database, the CIF files of nepheline, sodalite, natrolite, analcime, and albite were downloaded and imported into Materials Studio. The MaterialsVisualizer module was used to adjust the automatically generated model (such as building chemical bonds and removing unnecessary water molecules) to establish the initial N-A-S-H model.

(2) The initial model was supercellulated by 2a × 2b × 2c to facilitate the subsequent structural optimization and water molecule adsorption calculation. The model was orthogonalized, and the angle γ in the box parameter was adjusted to 90° after supercellulation, with the box size appropriately expanded.

(3) The model’s structure optimization and dynamic relaxation are conducted using the Forcite module. The specific operations are as follows: The Geometry Optimization function is chosen to optimize the structure, with Universal, ClayFF, and COMPASS III selected as the force fields. Fine is selected as the optimization mass, Smart as the method, Energy as 0.0001 kcal/mol, Force as 0.005 kcal/(mol·Å), Stress as 0.005 GPa, and Charges as Forcefield assigned. For electrostatic and van der Waals interactions, Ewald is selected. The Dynamics function is chosen for dynamic relaxation, with Fine selected for model optimization quality. This includes an initial temperature of 300 K, a rising temperature of 500 K, running 50,000 steps, NVT ensemble (300 K, 0 Pa), and Nose-Hoover thermostat method. The remaining parameter settings are the same as during energy minimization.

(4) A water molecule model is established separately, and the structure optimization and energy optimization of the water molecule model are carried out using the Forcite module. The Monte Carlo (GCMC) method is used to adsorb the appropriate water molecules. The parameters are set as follows: select the sorption tool, choose the optimized water molecule model for the adsorbate, select the task item select the quality simulate a maximum loading step of 500,000 steps, process step of 100,000 steps, temperature cycle four times, automatically control temperature, and automatically adjust the step size.

**Figure 4:**
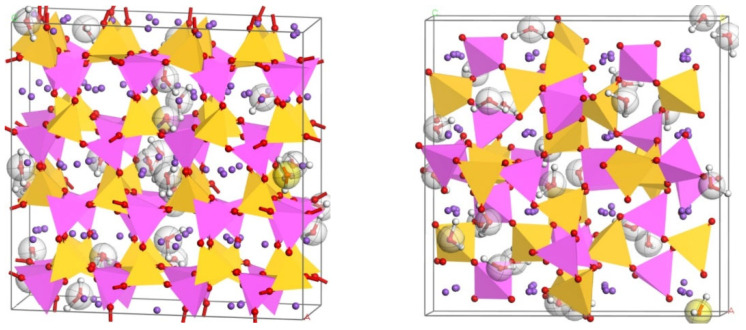
N-A-S-H model based on nephelite and sodalite under ClayFF force field (n(Si)/n(Al) = 1).

**Figure 5:**
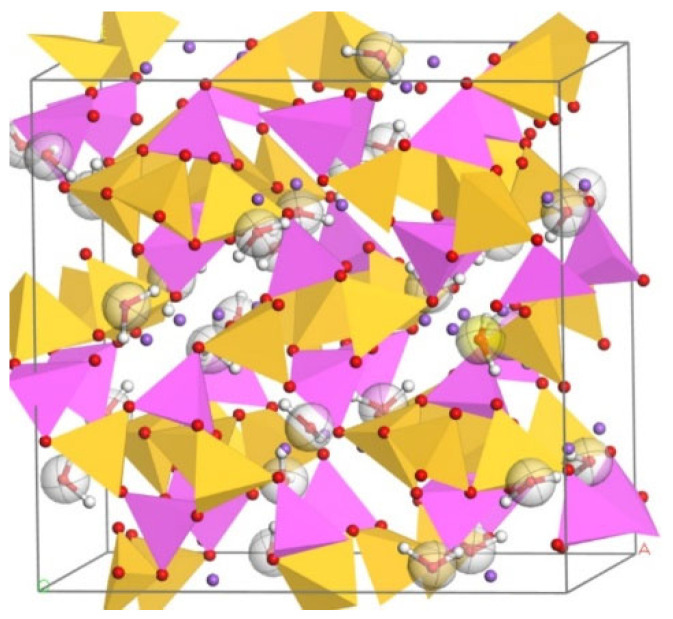
N-A-S-H model based on natrolite under ClayFF force field ClayFF (n(Si)/n(Al) = 1.5).

**Figure 6:**
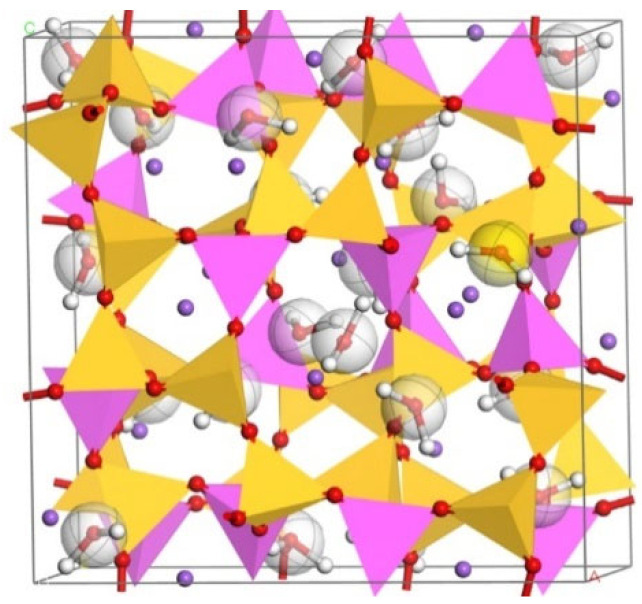
N-A-S-H model based on analcime under ClayFF force field (n(Si)/n(Al) = 2).

**Figure 7:**
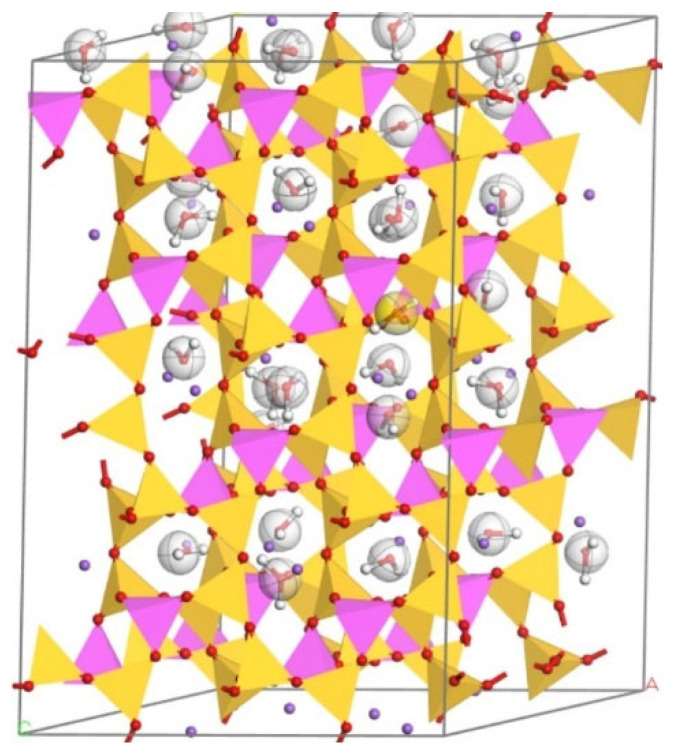
N-A-S-H model based on albite under ClayFF force field (n(Si)/n(Al) = 3).

The results of various n(Si)/n(Al) models under the ClayFF force field are shown in Figure 4, Figure 5, Figure 6 and Figure 7.

### 3.3. Model Reliability Analysis Under Different Force Fields

In MaterialsStudio 7.0, there is currently no force field for geopolymers or inorganic polymers to choose from, but the coverage of the following three force fields covers zeolite calculations. Universal has full coverage of the periodic table and contains the zeolite calculation of common silicon, aluminum, and oxygen atomic force field forms. It is explained in the software operation manual. Universal is moderately accurate for predicting geometries and conformational energy differences of organic molecules, main-group inorganics, and metal complexes. COMPASS (Condensed-phase Optimized Molecular Potentials for Atomistic Simulation Studies) is a high-quality force field to consolidate parameters of organic and inorganic materials. COMPASS III is widely used in polymer and inorganic materials. Based on the basic force field, the parameters of COMPASS III are optimized using density functional theory, which makes the calculation results more accurate. ClayFF does not belong to the initial force field file of MaterialsStudio 7.0, but its force field information can be found and imported into the force field file of MS. This position is a general position, widely used in the calculation of C-S-H due to its applicability to multi-component minerals and corresponding hydroxides.

**Figure 8:**
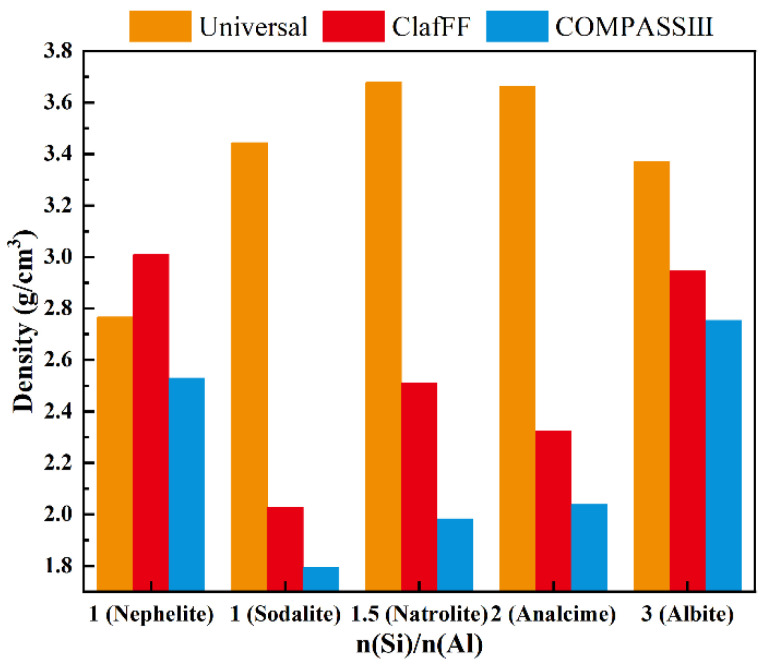
The density of N-A-S-H model under three different force fields.

**Figure 9:**
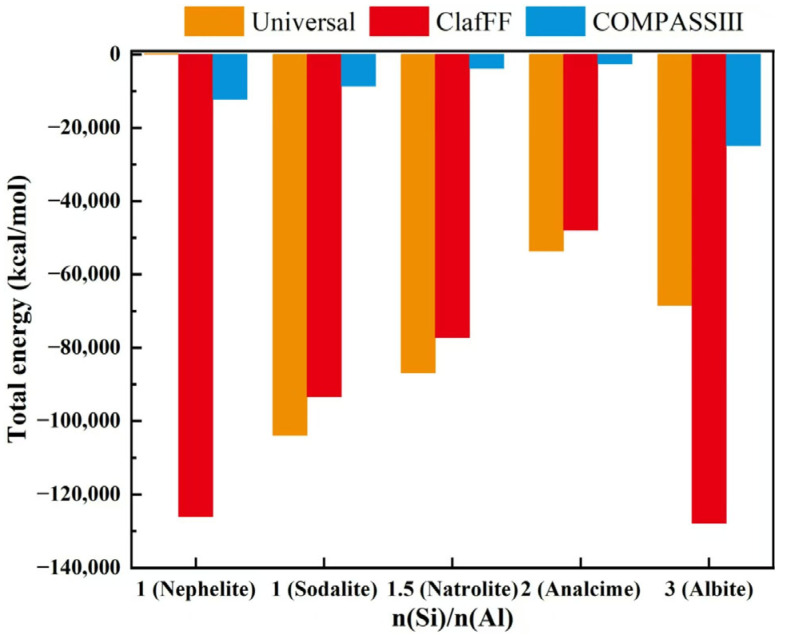
The total energy of N-A-S-H model under three different force fields.

**Figure 10:**
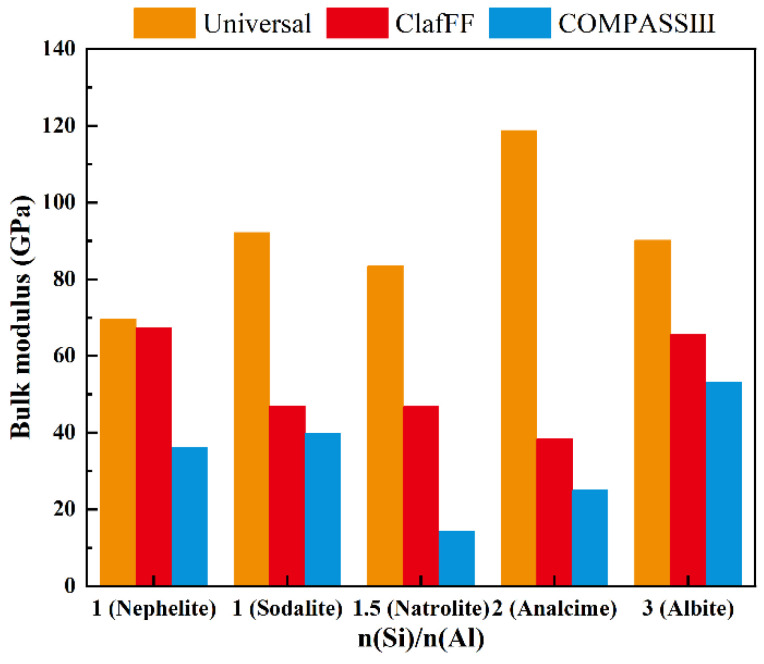
The bulk modulus of N-A-S-H model under three different force fields.

The density (Figure 8), energy (Figure 9), and bulk modulus (Figure 10) of the model are calculated under three different force fields, and the calculation results show significant differences. The material density calculated by the Universal force field is high, which differs greatly from the actual scenario. The calculation results of the other two force fields closely follow the calculation trend, both showing ‘W’ type broken lines. The nephelite model has a higher density due to its closer stacking. The other four models have the same stacking mode (ABC stacking). Under the two force fields, the overall density increases with the increase of n(Si)/n(Al). An exception is that the analcime model has a lower density than the natrolite model under the ClayFF force field. The energy calculation results effectively demonstrate the applicability of the three different force fields. The energy calculation results of various models under the COMPASS III force field are stable and consistently maintained at the same order of magnitude. The absolute energy value of the analcime model is the lowest, while the albite model has the highest absolute energy value. Although the calculated results of the ClayFF force field fluctuate significantly, the overall trend aligns with the results of COMPASS III. The nephelite model energy calculated by Universal is positive, but the absolute energy value of the N-A-S-H model of the ABC-6 family type also shows a trend of initially increasing and then decreasing with the increase of n(Si)/n(Al), with the lowest point being the analcime model. The other four models have the same stacking mode (ABC stacking). Under the two force fields, the overall density increases with the increase of n(Si)/n(Al). An exception is that the analcime model has a lower density than the natrolite model under the ClayFF force field.

The energy calculation results effectively demonstrate the applicability of the three different force fields. The energy calculation results of various models under the COMPASS III force field are stable and consistently maintained at the same order of magnitude. The absolute energy value of the analcime model is the lowest, while the albite model has the highest absolute energy value. Although the calculated results of the ClayFF force field fluctuate significantly, the overall trend aligns with the results of COMPASS III. The nephelite model energy calculated by Universal is positive, but the absolute energy value of the N-A-S-H model of the ABC-6 family type also shows a trend of initially increasing and then decreasing with the increase of n(Si)/n(Al), with the lowest point being the analcime model. The stability of energy values across various models using the COMPASS III force field suggests that this force field provides a consistent description of the potential energy landscape for the N-A-S-H structures. This consistency implies that COMPASS III accurately captures the fundamental interactions that govern the stability of these structures. The lower energy value observed for the analcime model may indicate that this structure represents a more energetically favorable configuration, potentially due to its unique arrangement of Si and Al atoms that minimizes strain within the framework. Conversely, the higher energy value for the albite model could suggest that this configuration is less stable and may be more prone to structural rearrangements, which could impact the long-term durability of geopolymers based on this structure.

The calculation results of the bulk modulus are shown in the Figure 10. The mechanical properties at the molecular scale are quite different from the macroscopic mechanical properties. The synchrotron radiation high-pressure X-ray diffraction technique is an effective tool to test the mechanical properties at the molecular scale. Jae et al. [32] performed synchrotron radiation high-pressure XRD tests on the ideal N-A-S-H similar to basic sodalite and measured its bulk modulus to be 43 ± 4 GPa. In the subsequent measurement based on the same method, the ideal N-A-S-H bulk modulus of approximately hydroxycancrinite was measured to be 46 ± 5 MPa [35], and it was considered that all zeolitic materials encapsulate singlenite six-membered rings (i.e., hydroxycancrinite, sodalite, and nepheline) have similar bulk moduli. The variation in bulk modulus as determined by different force fields underscores the sensitivity of this mechanical property to the specific atomic interactions and structural arrangements within the N-A-S-H framework. The close agreement between the COMPASS III force field results and experimental data suggests that this force field effectively captures the intricate balance of forces within the N-A-S-H structure, which is critical for accurately predicting bulk modulus. The calculation results of ClayFF force field and Universal force field are quite different. The lower bulk modulus of the natrolite model, as indicated by the COMPASS III calculations, could be attributed to the structural flexibility inherent in its framework. This flexibility may arise from the natrolite’s specific n(Si)/n(Al) arrangement, which allows for greater ease in deforming under pressure, thus contributing to its lower bulk modulus. This kind of geopolymer has relatively good compression space and flexibility. The bulk modulus on both sides is improved, and the rigidity of the geopolymer is enhanced. This characteristic could be advantageous in applications where some degree of compressibility is desirable.

In summary, for the construction of the N-A-S-H model, the Compass III is the most suitable, followed by the ClayFF, while the Universal is not recommended for N-A-S-H model calculations. The calculations using the COMPASS III force field indicate that the theoretical density of the N-A-S-H model increases with the ratio of n(Si)/n(Al). Moreover, for N-A-S-H models with different stacking modes, their density is higher than that of the Sodalite model with the same n(Si)/n(Al). The total energy of each model remains within a similar range. Under the same stacking mode, the total energy initially decreases and then increases, with the lowest and most stable energy observed in the analcime N-A-S-H model. Conversely, for different stacking modes of the N-A-S-H model, the energy is higher than that of the sodalite model. The bulk modulus of the N-A-S-H model with the same stacking mode decreases and then increases with the n(Si)/n(Al), with the natrolite-type N-A-S-H model having the lowest bulk modulus. For different stacking modes of the N-A-S-H model, the bulk modulus based on nephelite is smaller than that of the Sodalite-type N-A-S-H model.

## 4. Conclusions

This study provides important insights into the structure and properties of geopolymers by tuning the n(Si)/n(Al) ratio. The results were confirmed by XRD analysis and molecular dynamics simulations, and help to deepen the understanding of the microstructure of geopolymers and its impact on material properties. These analyses led to the following conclusions:

(1) The N-A-S-H structure of the geopolymer with n(Si)/n(Al) = 1 is closest to the sodalite structure. The N-A-S-H structure of the geopolymer with n(Si)/n(Al) = 1.5 is closest to the natrolite structure, and the N-A-S-H structure of the geopolymer with n(Si)/n(Al) = 2 is closest to the analcime structure. The N-A-S-H structure of the geopolymer with n(Si)/n(Al) = 3 is closest to the albite structure.

(2) N-A-S-H model building and molecular dynamics simulations are conducted using MaterialsStudio 7.0. Based on the results of density calculation, energy calculation, and bulk modulus calculation, the Compass Ⅲ is found to be the most suitable for N-A-S-H, followed by the ClayFF. The Universal is deemed unsuitable.

(3) According to the calculation results of Compass III, the theoretical density of the N-A-S-H model based on the same stacking mode increases with the increase of n(Si)/n(Al). The absolute value of total energy decreases initially and then increases, while the absolute value of energy of the analcime N-A-S-H model is the lowest and is relatively stable. The change in bulk modulus decreases initially and then increases with the increase of n(Si)/n(Al). The lowest point of bulk modulus is the natrolite type N-A-S-H model.

## Figures and Tables

**Table 1:** XRF analysis of MK and NS.

Oxide (wt%)	SiO_2_	Al_2_O_3_	MgO	Fe_2_O_3_	CaO	Others
MK	38.63	54.22	1.60	1.53	1.13	2.89
NS	98.80					1.20

**Table 2:** Composition ratio of the sample.

Sample	MK (wt%)	NS (wt%)	n(Si)/n(Al)
GEO-1	100	0	0.6
GEO-2	63	37	1.5
GEO-3	53	47	2.0
GEO-4	45	55	2.5

## Data Availability

The original contributions presented in the study are included in the article, further inquiries can be directed to the corresponding author.
